# Trends in tooth loss among adults and older adults in municipalities participating in oral national surveys 2003, 2010 and 2023

**DOI:** 10.1590/1980-549720260030.supl.1

**Published:** 2026-08-24

**Authors:** Sônia Cristina Lima Chaves, Jony Arrais Pinto, Thais Regis Aranha Rossi, Thamyres Pereira Lima, Ana Tereza Ribeiro da Silva, Ana Maria Freire de Souza Lima, Joana Danielle Brandão Carneiro, Doralice Severo da Cruz Teixeira, Sandra Garrido de Barros, Leonardo Marchini, Monira Samann Kallás

**Affiliations:** I Universidade Federal da Bahia, Institute of Collective Health – Salvador (BA), Brazil.; II Universidade Federal Fluminense – Rio de Janeiro (RJ), Brazil.; III Universidade do Estado da Bahia, Institute of Collective Health – Salvador (BA), Brazil.; IV Secretaria de Saúde do estado da Bahia – Salvador (BA), Brazil.; V Universidade Federal da Bahia – Salvador (BA), Brazil.; VI Ministério da Saúde – Brasília (DF), Brazil.; VII Universidade Federal da Bahia, School of Dentistry – Salvador (BA), Brazil.; VIII University of Iowa, College of Dentistry and Dental Clinics – Iowa City, Iowa, United States of America.; IX Hospital Sírio Libanês – São Paulo (SP), Brazil.

**Keywords:** Tooth loss, Oral health care, Health policy, Dental caries, Aging

## Abstract

**Objective:**

This study analyzed trends in tooth loss and caries severity among adults and older adults from 2003 to 2023.

**Methods:**

An ecological study using secondary data from the three national oral health Surveys (SB Brasil 2003, 2010, and 2023) in 43 municipalities that participated in all editions. The analysis of trends in caries severity and tooth loss among adults (35–44 years) and older adults (65–74 years) was performed using structural equation modeling.

**Results:**

Among adults, a significant association was observed between higher illiteracy rates and increased the decayed, missing, and filled teeth index (DMFT) (β=+0.309; p<0.001). On the other hand, greater coverage by oral health teams and private dental plans were associated with a reduction in these outcomes (β=-0.040 p<0.001 and β=-5.527 p<0.001, respectively). The associations were less evident but pointed in the same direction among older adults regarding illiteracy rates and private dental plan coverage, where the estimated coefficients showed lower magnitude and significance, indicating moderation of contextual effects and stability of the indicators over time.

**Conclusion:**

The study confirmed a downward trend in tooth loss and DMFT between 2003 and 2023, pronounced in adults and to a lesser extent in older adults in Brazil. The latter are particularly vulnerable to rapid deterioration in oral health, requiring individualized analyses in future studies and the targeting of efforts by policymakers and implementers.

## INTRODUCTION

Tooth loss represents the outcome of untreated oral diseases, particularly dental caries and periodontal disease^1^. Despite the global decline in the prevalence of severe tooth loss, it remains a public health issue and part of a significant global burden. It is estimated that approximately 23% of people aged 60 and older are completely edentulous^2,3^. This burden is unevenly distributed worldwide, with higher prevalence observed in low-income regions^4,5^.

Nevertheless, recent decades have revealed significant changes in patterns of dental caries and tooth loss among adults and older people, with marked variations between high-, middle-, and low-income countries. In developed countries, there has been a significant decline in the prevalence and severity of caries and tooth loss. Tooth retention has increased, but this has led to a relative rise in caries among elderly individuals, as more natural teeth endure^4,6^.

High rates of caries and tooth loss persist, with inequalities exacerbated by socioeconomic factors, limited access to preventive care, and a predominance of tooth extractions over restorative treatments in low- and middle-income countries. The negative impact on quality of life is more pronounced in these contexts^4-6^. In the case of Brazil, the conduct of regular epidemiological surveys^7-9^ can serve as an analytical tool, like retrospective cohort models. Cross-sectional studies and population cohorts are the strategies yielding most of the data^10^, with an absolute predominance of the DMFT index (decayed, missing, and filled teeth) and World Health Organization (WHO) criteria for diagnosing caries and tooth loss^4,7-9^.

Ferreira et al.^11^ investigated the evolution of dental caries and edentulism among older Brazilian adults between 2003 and 2023, noting that the mean DMFT index fell from 27.6 to 23.5 and edentulism, total tooth loss, dropped from approximately 53 to 36%, with these improvements concentrated among white individuals and those with higher levels of education^11^. However, few studies provide aggregated analyses that incorporate health service variables and trend differences during life, from adulthood to old age^6^, while comparing these two groups. In this context, the present study aimed to analyze trends in tooth loss and dental caries severity among adults and older adults in the municipalities participating in the three national oral health surveys (SB Brasil 2003, 2010, and 2023), using a strategy of intra-municipal analysis and control for contextual variables over time. Furthermore, it sought to identify differences between age groups and associated socioeconomic and health service variables.

## METHODS

This was an ecological study with a quantitative approach, based on secondary data from the SB Brasil surveys of 2003, 2010, and 2023^7-9^. The analysis included the 43 municipalities that participated in all editions of the survey, ensuring temporal comparability (Chart 1). Sample size was determined by the availability of data from the national oral health surveys conducted during the analyzed period, resulting in the inclusion of municipalities with complete and comparable information over time.

**Chart 1:** Municipalities selected for the study that participated in the epidemiological surveys in 2003, 2010, and 2023.

Capitals	Large municipalities in 2023 (federative unit)	Medium-sized municipalities in 2023 (federative unit)
Aracaju (SE) Belém (PA) Belo Horizonte (MG) Boa Vista (RR) Brasília (DF) Campo Grande (MS) Cuiabá (MT) Curitiba (PR) Florianópolis (SC) Fortaleza (CE) Goiânia (GO) João Pessoa (PB) Macapá (AP) Maceió (AL) Manaus (AM) Natal (RN) Palmas (TO) Porto Alegre (RS0 Porto Velho (RO) Recife (PE) Rio Branco (AC) Rio de Janeiro (RJ) Salvador (BA) São Luís (MA) São Paulo (SP) Terezina (PI) Vitória (ES)	Ananindeua (PA) Aparecida de Goiânia (GO) Blumenau (SC) Cametá (PA) Duque de Caxias (RJ) Itajaí (SC) Luziânia (GO) Parauapebas (PA) Santarém (PA) Várzea Grande (MT)	Anápolis (GO) Cáceres (MT) Corumbá (MS) Cruzeiro do Sul (AC) Parintins (AM) Tefé (AM)

Small-sized: 5,001 to 20,000 inhabitants; medium-sized: 20,001 to 100,000 inhabitants; large-sized: over 100,000 inhabitants.

Source: Data from SB Brasil 2003, 2010, and 2023.

The study focuses on the severity of dental caries and tooth loss. The outcomes considered were the municipal mean DMFT index scores (mean number of decayed, missing, and filled teeth per individual) and specifically the "M" component, representing missing teeth. Analyses were stratified by age group, covering adults (35–44 years) and older adults (65–74 years). The percentage contribution of the "M" component to the DMFT index was also analyzed. The national datasets used originate from the SB Brasil 2003, 2010, and 2023 surveys; the results are currently available as a public database with free access. It should be noted that the data from the SB Brasil 2003, 2010, and 2023 surveys derive from complex, independent sampling designs. In the present study, outcomes were operationalized as proportions aggregated by municipality. Since individual sample weights are not available for the SB Brasil 2003 survey, an ecological and comparative approach was adopted, without individual weighting.

To assess trends and patterns of variability in the outcomes, spaghetti plots were created, accompanied by a mean curve representing the general trend across the three years evaluated. For each outcome and year, the mean, standard deviation, minimum and maximum values, and the 95% confidence interval of the mean were presented. The following were considered covariates: illiteracy rate (%), population coverage by oral health teams (OHT) within the Unified Health System (SUS), private dental plan coverage (% of the population with insurance), and the municipality's status (capital or non-capital).

Data regarding OHT coverage (40-hour teams, including Quilombola- and settlement-based OHT, as well as modalities I and II) for 2003, 2010, and 2023 were collected from the eGestor Primary Care website (https://egestorab.saude.gov.br). The coverage rate for dental-only health plans was obtained via the TabNet system of the National Supplementary Health Agency (ANS) at https://www.ans.gov.br/anstabnet/cgi-bin/dh?dados/tabnet_tx.def.

The illiteracy rate for the population aged 15 and older was obtained from the Human Development Atlas in Brazil (Atlas DH), based on microdata from the Brazilian Institute of Geography and Statistics (IBGE) Demographic Censuses. Data from the 2000, 2010, and 2022 Demographic Censuses were used, sourced from https://www.ipeadata.gov.br/Default.aspx, corresponding to the years closest to the analyzed surveys (2003, 2010, and 2023).

To evaluate the associations between these explanatory variables and the outcomes at three distinct time points (2003, 2010, and 2023) within each age stratum, generalized estimating equation (GEE) models were fitted, as proposed by Liang and Zeger^12^.

The models were specified with an identity link function and a normal (Gaussian) distribution, which are appropriate for continuous outcomes. Different working correlation structures were tested (independence, AR(1), unstructured, and exchangeable); the exchangeable structure was selected because it assumes constant correlation over time and offers superior numerical stability and convergence. Estimator variances were obtained using the robust sandwich estimator, ensuring consistent inference even in the event of correlation misspecification. All analyses were conducted using R software (version 4.4.3) (R Core Team, 2025), adopting a 5% significance level^13^. The study report followed the recommendations of the STROBE (Strengthening the Reporting of Observational Studies in Epidemiology) checklist.

### Data availability statement:

The entire dataset supporting the results of this study is available upon request from the corresponding author. The dataset is not publicly available due to database integration performed by the authors.

## RESULTS

**Figure 1:**
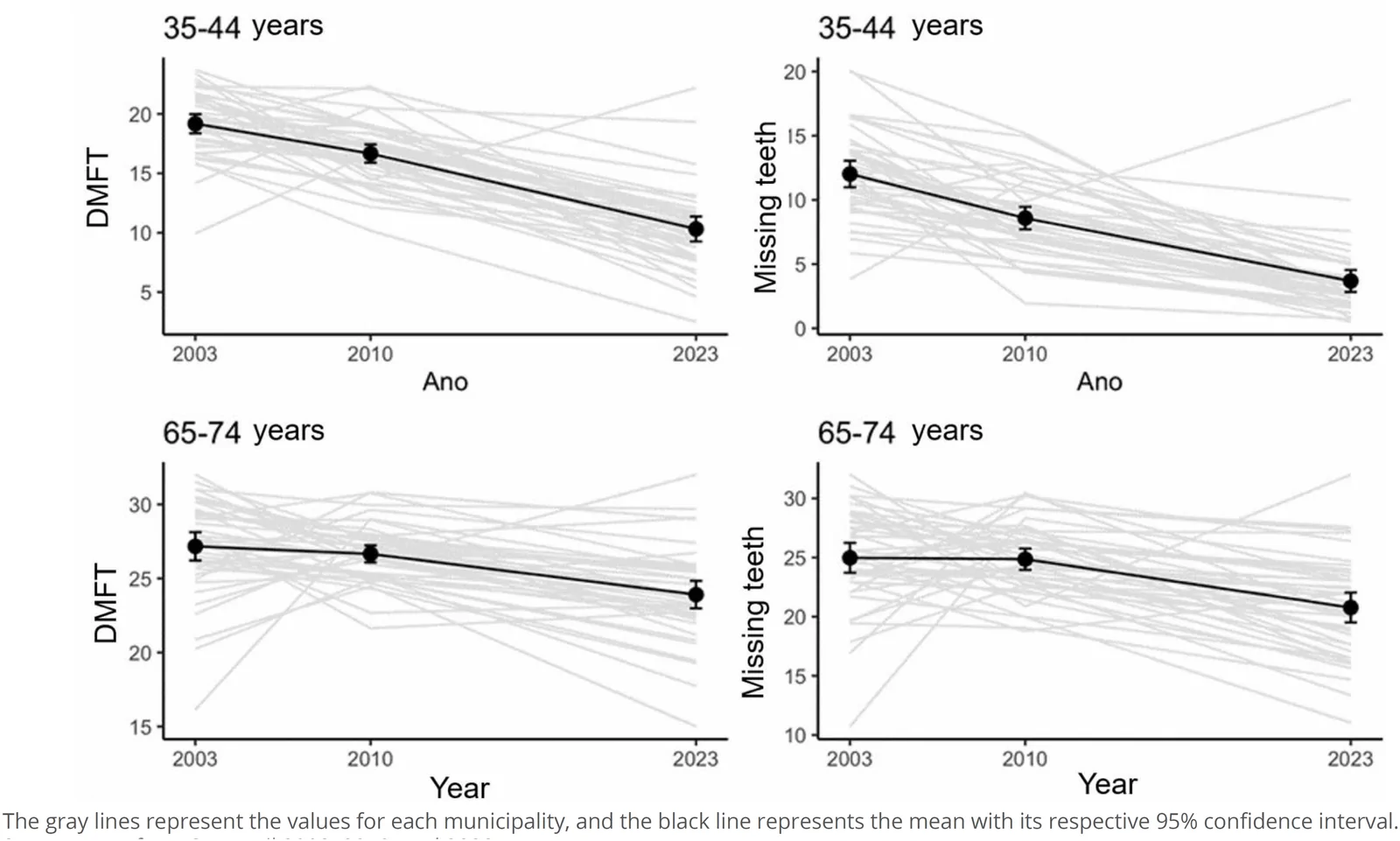
Trends in mean DMFT scores and the "M" (missing teeth) component in 2003, 2010, and 2023, by age groups 35–44 years (top panels) and 65–74 years (bottom panels), among municipalities participating in the three editions of the National Oral Health Survey. (n=43).

Source: Data from SB Brasil 2003, 2010, and 2023.

Across the 43 municipalities analyzed, the decrease in DMFT as well as its "Missing" component was significant during the period. However, two municipalities showed a divergent trend: Santarém (Pará) and Cáceres (Mato Grosso), where this indicator increased and stabilized among adults, respectively (Chart 1). Among the older individuals, only Aparecida de Goiânia (in the Central-West region) also exhibited a divergent upward trend compared to the other municipalities. Visual analysis of the trend curve reveals that the decline was greater among adults than older people (Figure 1).

The longitudinal analysis revealed a downward trend in oral health indicators among adults between 2003 and 2023 (Table 1). In this 35–44 age group, the mean DMFT index decreased from 19.17 (95%CI 18.37–19.98) in 2003 to 16.67 (95%CI 15.90–17.43) in 2010, and 10.32 (95%CI 9.27–11.37) in 2023, indicating successive reductions and non-overlapping confidence intervals across the three periods. The "M" component (missing teeth) showed a similar pattern, declining from 12.02 (95%CI 10.99–13.05) in 2003 to 8.58 (95%CI 7.71–9.46) in 2010, and 3.69 (95%CI 2.83–4.54) in 2023, representing a cumulative drop of over 65% during the period (Table 1).

**Table 1:** Mean, standard deviation, and 95% confidence interval for DMFT, the "M" component (missing teeth), and the percentage of missing teeth in the DMFT index among adults (35–44 years old) and older adults (65–74 years old) in municipalities monitored across the three editions of the SB Brasil survey. (2003, 2010, and 2023).

Year		Mean	SD	%M in DMFT	Minimum	Maximum	95%CI
DMFT 35–44 years							
	2003	19.17	2.70	-	9.94	23.69	18.37–19.98
	2010	16.67	2.56	-	10.17	22.33	15.90–17.43
	2023	10.32	3.51	-	2.50	22.20	9.27–11.37
Missing teeth 35–44 years							
	2003	12.02	3.45	62.70	3.82	20.11	10.99–13.05
	2010	8.58	2.93	51.47	1.95	15.17	7.71–9.46
	2023	3.69	2.87	35.76	0.50	17.80	2.83–4.54
DMFT 65–74 years							
	2003	27.17	3.20	-	16.14	32.0	26.21–28.12
	2010	26.66	1.92	-	21.63	30.8	26.08–27.23
	2023	23.91	3.11	-	15.00	32.0	22.98–24.84
Missing teeth 65–74 years							
	2003	24.98	4.22	91.94	10.71	32.00	23.71–26.24
	2010	24.86	3.00	93.25	18.78	30.44	23.97–25.76
	2023	20.77	4.22	86.87	11.02	32.00	19.51–22.03

Source: SB Brasil 2003, 2010, and 2023.

Among older adults aged 65 to 74, stability was observed between 2003 and 2010 for both the DMFT index and the "M" (missing) component, with widely overlapping confidence intervals (DMFT: 27.17 [95%CI 26.21–28.12] in 2003 and 26.66 [95%CI 26.08–27.23] in 2010; "M": 24.98 [95%CI 23.71–26.24] and 24.86 [95%CI 23.97–25.76], respectively). A significant decline was noted only in 2023, with mean values of 23.91 (95%CI 22.98–24.84) for DMFT and 20.77 (95%CI 19.51–22.03) for the "M" component (Table 1). These results indicate consistent progress in tooth retention over the two decades, although the rate of improvement was greater among adults. Regarding the proportional contribution of missing teeth to the DMFT index, a sharp decrease was observed in the adult group between 2003 and 2023, whereas the reduction among older adults was not of the same magnitude. "M" accounted for 62.7% of the DMFT index in 2003, going to 35.76% in 2023. Among older adults, however, it represented 91.94% of the DMFT index in 2003 and decreased only slightly to 86.87% in 2023 (Table 1).

GEE modeling allowed for the assessment of the influence of contextual factors on oral health outcomes, accounting for within-municipality correlation over time. Among adults (35–44 years old), the model for the DMFT index indicated a positive association between the illiteracy rate and the mean index value (β=0.309; p<0.001), revealing that municipalities with a higher proportion of illiterate individuals exhibited worse DMFT indicators. Conversely, OHT coverage showed a significant negative association (β=-0.040; p=0.011), suggesting a protective effect of expanding primary oral health care against the severity of caries and tooth loss among adults. Private dental plan coverage also showed a significant negative association (β=-4.527; p<0.001), indicating that greater access to private dental services is associated with lower mean DMFT values (Table 2).

**Table 2:** Results of GEE models for factors associated with the downward trend in DMFT and mean number of missing teeth among adults (35–44 years) and older adults (65–74 years) in municipalities (n=43) participating in all National Oral Health Surveys (SB Brasil 2003, 2010, and 2023).

Variable		Estimated association (β)	Standard error	Test statistic	p-value
DMFT 35–44 years					
	(Intercept)	15.350	1.341	130.994	<0.001
	Illiteracy	**0.309**	**0.084**	**13.371**	**<0.001**
	Cov_OHT	**-0.040**	**0.011**	**12.146**	**<0.001**
	Cov_pr.plan	**-4.527**	**0.640**	**49.980**	**<0.001**
	Capital	1.541	0.980	2.476	0.116
Missing teeth 35–44 years					
	(Intercept)	6.976	1.209	33.282	<0.001
	Illiteracy	**0.507**	**0.079**	**41.555**	**<0.001**
	Cov_OHT	**-0.046**	**0.011**	**16.747**	**<0.001**
	Cov_pr. Plan	**-3.574**	**0.469**	**58.104**	**<0.001**
	Capital	0.968	0.853	1.287	0.257
DMFT 65–74 years					
	(Intercept)	26.863	0.822	928.238	<0.001
	Illiteracy	0.043	0.066	0.432	0.511
	Cov_OHT	-0.017	0.010	2.611	0.106
	Cov_pr.plan	**-2.586**	**0.447**	**33.476**	**<0.001**
	Capital	0.404	0.706	0.327	0.567
Missing teeth 65–74 years					
	(Intercept)	24.665	1.009	586.185	<0.001
	Illiteracy	0.063	0.081	0.605	0.437
	Cov_OHT	-0.013	0.013	0.924	0.336
	Cov_pr.plan	**-3.766**	**0.758**	**24.705**	**<0.001**
	Capital	0.356	0.845	0.178	0.673

Statistical significance indicated in bold. cov_OHT, oral health team coverage; cov_pr.plan, private dental plan coverage.

Source: SB Brasil 2003, 2010, and 2023.

A similar pattern was observed for the "M" component (missing teeth) in the same age group. The illiteracy rate showed a significant positive association (β=0.507; p<0.001), reinforcing the role of educational attainment in oral health inequalities. Oral health team coverage maintained a negative association (β=-0.046; p<0.001), indicating that higher coverage is linked to a reduction in the mean number of missing teeth. Private dental plan coverage also showed a significant negative association (β=-3.574; p<0.001), suggesting that access to private dental services further contributes to lower average tooth loss among individuals aged 35–44. Overall, the study indicates that lower educational levels and lower public and private coverage are associated with poorer outcomes, whereas the expansion of OHT exerts a protective effect in this group (Table 2).

Among the older adults (65–74 years old), the estimated coefficients showed lower magnitude and significance, indicating an attenuation of contextual effects and greater stability of the indicators over time. Regarding the DMFT index, illiteracy was not significantly associated with the outcome (β=0.043; p=0.511), and OHT coverage showed a non-significant negative association (β=-0.017; p=0.106). In contrast, private dental plan coverage showed a significant association (β=-2.586; p<0.001). Similar results were observed for the "M" (missing teeth) component, where the illiteracy rate was not significantly associated with the mean number of missing teeth (β=0.063; p=0.437), while OHT coverage maintained a non-significant negative association (β=-0.013; p=0.336). On the other hand, private dental plan coverage maintained a negative and statistically significant association with the mean number of missing teeth (β=-3.766; p<0.001). Capital city status did not influence any of the adjusted models (Table 2).

## DISCUSSION

This study confirmed the trend of a significant decline in tooth loss and DMFT scores between 2003 and 2023 — a decline that was more pronounced between 2010 and 2023, particularly among the adult population. It highlights the protective role of education, the expansion of OHT coverage, and access to private dental plans within this group. Socioeconomic inequalities, as reflected by illiteracy rates, continue to exert a significant influence on oral health outcomes, especially among younger age groups.

Among the older individuals, illiteracy rates showed no statistically significant effect, suggesting a need for further investigation into the cumulative impacts over the passage of time. It is suggested that characteristics associated with this stage of life and the history of healthcare access tend to outweigh current educational status. Other potential explanations relate to the nature of aging in Brazil, which is marked by severe inequalities, namely, psychological, physical, and mental^14^.

Studies have been conducted to explore the specific characteristics of tooth loss among older people in Brazil and other countries^15-17^. It is known that older adults are particularly vulnerable to rapid oral health deterioration (ROHD) because of factors such as chronic comorbidities, polypharmacy, extensive dental restorations, and age-related socioeconomic challenges^15^.

Tooth loss, particularly total edentulism, is a robust indicator of overall oral health status and remains a significant global public health issue, especially among the elderly population^18^. Tooth loss is not limited to the oral cavity; it has significant repercussions for systemic health, particularly in older adults, and is associated with an increased incidence of geriatric syndromes. Edentulous individuals or those with partial tooth loss are more likely to be malnourished or at risk of malnutrition^19^.

It is worth noting that population aging is a global, multifaceted phenomenon resulting directly from increased life expectancy and declining fertility rates^20^. While representing a historic achievement for humanity, this process brings complex challenges for healthcare, social security, and social welfare systems. The implications of aging are particularly evident in oral health, as oral diseases, which accumulate over a lifetime, frequently lead to tooth loss; this, in turn, compromises essential functions such as chewing, swallowing, and speech, while also impacting individuals’ aesthetics and self-esteem^20-23^. Although age-standardized prevalence has declined in many developed countries over recent decades, the absolute growth of the elderly population has led to an increase in the total number of people with severe tooth loss, as demonstrated by Global Burden of Disease data from 1990 to 2019^20^.

As with the study by Ferreira et al.^11^, the present study found a significant reduction in the number of lost teeth between 2003 and 2023, a trend not observed between 2003 and 2010. In other words, the data confirmed the analysis of the aggregate of the 43 municipalities included in this study. However, it is worth noting that this reduction was unequal, primarily benefiting White individuals and those with higher levels of education^11^; indeed, factors such as low education, poverty, chronic comorbidities, smoking, and poor diet have been identified as risk determinants for tooth loss in multicenter studies involving countries such as China, India, Mexico, and South Africa^23^.

This scenario underscores the urgent need for public policies that integrate oral health into care models focused on aging, as tooth loss should not be viewed as an inevitable consequence of growing older. The global average prevalence of tooth loss is 7% (350 million people)^24^, highlighting the need for preventive interventions starting in early adulthood, as well as health promotion initiatives aimed at reducing tooth loss in older people^25^. Studies acknowledge the importance of primary health care in oral health^25,26^, as increased OHT coverage within the Family Health Strategy model has been linked to a reduction in tooth loss indicator, in line with the present finding of a reduction in the "M" (missing/lost) component specifically among adults. Nevertheless, research has shown that cities with lower human development index scores and lower OHT coverage experienced higher rates of tooth extractions^27^. A recent initiative to introduce an indicator encouraging a reduction in the percentage of extractions relative to other primary care procedures aims to intervene effectively at the early stages of the natural history of diseases (such as caries and periodontal disease), prioritizing less mutilating procedures (both preventive and curative) over extractions^28^.

Factors associated with better oral health in older adults include regular access to dental services, and the protective effect of insurance is stronger among those with higher socioeconomic status and healthier lifestyles. The present study indicated a positive effect among older adults with private dental insurance regarding lower rates of tooth loss. However, having a private dental plan does not eliminate disparities on its own; education, income, and health behaviors remain important determinants^29^.

Furthermore, a recent study found that the absence of a dental plan was significantly associated with a higher prevalence of edentulism, even after adjusting for age, education, and lifestyle^30^. The lack of private dental insurance likely reflects both lower purchasing power and reduced access to preventive and restorative dental care, thereby increasing the risk of tooth loss^31^.

A cross-sectional study using the same population base as the current study revealed a high prevalence of total edentulism among older Brazilians (31.7%), highlighting a significant public health issue with aesthetic, functional, and psychosocial repercussions. A multivariate analysis demonstrated that total tooth loss is associated with unfavorable socioeconomic conditions, low levels of education, and the absence of a dental plan, reflecting inequalities in access to prevention and treatment. Additionally, behavioral factors such as smoking, consumption of sugary beverages, and a sedentary lifestyle were significantly linked to the condition, reinforcing the multifactorial nature of edentulism^32^. These findings underscore the need for integrated public policies that promote healthy habits and expand access to dental care, particularly among the most vulnerable groups within the older population^17^.

The international report published in 2022 by the World Health Organization highlights the global relevance of oral health in aging, aligning with the United Nations (UN) "Decade of Healthy Aging (2021–2030)" initiative, which recognizes oral health as an essential indicator of general health in older adults. Oral diseases, which are chronic and cumulative in nature, show high prevalence in older age groups, particularly edentulism, which has a global average of 22.7% among people over 60, with significant regional variations (e.g., Europe shows the highest rates at 31.3%). Severe periodontal disease also peaks around age 60, and population aging tends to increase its global burden. The document further emphasizes that socioeconomic inequalities exacerbate tooth loss, disproportionately affecting frail and institutionalized older adults. Japan is cited as an example of effective policy with its "8020 Campaign", aimed at preserving 20 natural teeth by age 80, reinforcing that continuous prevention and lifelong oral care lead to better nutrition, autonomy, and social integration in old age^24,33^.

Therefore, studies incorporating systemic health, socioeconomic, and oral health indicators contribute to identifying, diagnosing, and treating conditions that can lead to tooth loss^16^. It is necessary to advance the analysis of factors driving ROHD^15,16^ associated with tooth loss among older Brazilians. Future studies of this nature could provide evidence-based insights to help policymakers, implementers, and Brazilian oral health professionals better detect and support those at risk of rapid oral health deterioration. In this regard, recommendations developed by the European College of Gerodontology emphasize that many healthcare professionals are unprepared to identify or manage oral health issues in older people and, furthermore, that structural barriers, including limited public coverage, a lack of integration between dentistry and medical care, and difficulties in accessing services, contribute to underdiagnosis and a lack of treatment^33^.

As this study utilized aggregated data, it did not allow for a more in-depth analysis of the elderly population's quality of life, which constitutes a limitation. Furthermore, data from the SB Brasil surveys (2003, 2010, and 2023) stem from complex and independent sampling designs.

In this study, outcomes were operationalized as proportions aggregated by municipality, and the analytical objective was to estimate relative temporal variations and differences between groups of municipalities. While it would have been possible to calculate weighted proportions based on individual weights, sample weights are unavailable for the SB Brasil 2003 survey. Consequently, an ecological and comparative approach was adopted, a decision acknowledged here as a methodological limitation. Another limitation was that the final sample comprised only municipalities included in all three surveys (n=43), resulting in an overrepresentation of state capitals and large municipalities. To mitigate potential imbalances, an indicator variable for "state capital" was included in the adjusted models. Further studies are warranted to examine the specific characteristics of older Brazilians associated with higher rates of tooth loss, as this group appears more resistant to the historical-social processes that have reduced the prevalence of this condition in other demographic groups.

This study confirmed a declining trend in the DMFT index and tooth loss among adults and older adults. Factors such as lower illiteracy rates, OHT coverage, and private dental insurance were associated with this decline among adults. Among older individuals, however, associations were less evident, significant only for private dental insurance coverage, suggesting an attenuation of contextual effects and stability in the indicators over time.
