# Supplementary material for: Trends in tooth loss among adults and older adults in municipalities participating in oral national surveys 2003, 2010 and 2023
Source: Rev Bras Epidemiol. 2026 Aug 24;29(Suppl 1):e260030supl1. doi: 10.1590/1980-549720260030.supl.1 (PMC13516018; doi:10.1590/1980-549720260030.supl.1)
Supplement: Translation

# Translation: Tendências da perda dentária entre adultos e idosos nos municípios participantes dos inquéritos nacionais 2003, 2010 e 2023

**DOI:** 10.1590/1980-549720260030.supl.1.2

**Authors:** Sônia Cristina Lima Chaves, Jony Arrais Pinto, Thais Regis Aranha Rossi, Thamyres Pereira Lima, Ana Tereza Ribeiro da Silva, Ana Maria Freire de Souza Lima, Joana Danielle Brandão Carneiro, Doralice Severo da Cruz Teixeira, Sandra Garrido de Barros, Leonardo Marchini, Monira Samann Kallás

**Affiliations:** I Universidade Federal da Bahia, Instituto de Saúde Coletiva – Salvador (BA), Brasil.; II Universidade Federal Fluminense – Rio de Janeiro (RJ), Brasil.; III Universidade do Estado da Bahia, Instituto de Saúde Coletiva – Salvador (BA), Brasil.; IV Secretaria de Saúde do estado da Bahia – Salvador (BA), Brasil.; V Universidade Federal da Bahia – Salvador (BA), Brasil.; VI Ministério da Saúde – Brasília (DF), Brasil.; VII Universidade Federal da Bahia, Faculdade de Odontologia – Salvador (BA), Brasil.; VIII University of Iowa, College of Dentistry and Dental Clinics – Iowa City, Iowa, United States of America.; IX Hospital Sírio Libanês – São Paulo (SP), Brasil.

**Keywords:** Perda dentária, Serviço de saúde bucal, Política de saúde, Cárie dentária, Envelhecimento

## Abstract

**Objetivo:**

Este estudo analisou as tendências da perda dentária e severidade da cárie em adultos e idosos, no período de 2003 a 2023.

**Métodos:**

Estudo ecológico com dados secundários dos três Inquéritos Nacionais de Saúde Bucal (SB Brasil 2003, 2010 e 2023), em 43 municípios que participaram de todas as edições. A análise das tendências de severidade da cárie e perda dentária em adultos (35-44 anos) e idosos (65-74 anos) foi realizada utilizando Modelos de Equações Estruturais (MEE).

**Resultados:**

Entre os adultos, observou-se associação significativa entre maior taxa de analfabetismo e aumento do índice CPOD (dentes cariados, perdidos e obturados) (β=+0,309; p<0,001). Por outro lado, maior cobertura de equipes de Saúde Bucal (eSB) e de planos odontológicos privados esteve relacionada à sua redução (β=-0,040; p<0,001; β=-5,527; p<0,001, respectivamente). As associações foram menos evidentes, mas na mesma direção entre os idosos para taxa de analfabetismo e posse de planos privados odontológicos, em que os coeficientes estimados apresentaram menor magnitude e significância, indicando atenuação dos efeitos contextuais e estabilidade dos indicadores ao longo do tempo.

**Conclusão:**

O estudo confirma uma tendência de declínio na perda dentária e no CPOD entre 2003 e 2023, mais acentuada em adultos e em menor magnitude em idosos no Brasil. Estes últimos são particularmente vulneráveis à rápida deterioração da saúde bucal, demandando análises individualizadas em estudos futuros e o direcionamento de esforços dos formuladores e implementadores de políticas de saúde bucal para enfrentar este desafio em todos os níveis de atenção.

## INTRODUÇÃO

A perda dentária representa o resultado final das doenças orais não tratadas, particularmente a cárie dentária e a doença periodontal^1^. Apesar do declínio mundial na prevalência de perda dentária grave, ela continua sendo um problema de saúde pública e parte de uma carga global significativa. Estima-se que cerca de 23% das pessoas com 60 anos ou mais sejam completamente desdentadas^2,3^. Esse fardo é distribuído de forma desigual pelo mundo, com maior prevalência observada em regiões de baixa renda^4,5^.

Ainda assim, as últimas décadas revelaram mudanças importantes nos padrões de cárie dentária e perda dental entre adultos e idosos, com variações marcantes entre países de alta, média e baixa renda. Em países desenvolvidos, o declínio tem sido significativo na prevalência e severidade da cárie e da perda dentária. A retenção dentária aumentou, mas isso levou a um aumento relativo de cárie em idosos, pois mais dentes naturais permanecem na cavidade bucal^4,6^.

Persistem altas taxas de cárie e perda dentária, com desigualdades acentuadas por fatores socioeconômicos, acesso limitado a cuidados preventivos e predominância de exodontias em relação aos tratamentos restauradores em países de baixa e média renda. O impacto negativo na qualidade de vida é mais pronunciado nestes contextos^4-6^. No caso do Brasil, a realização de inquéritos epidemiológicos regulares^7-9^ pode ser ferramenta de análise como modelos de coorte retrospectiva. Estudos transversais e coortes populacionais são estratégias das quais provém a maioria dos dados^10^, com predominância absoluta do índice CPOD (dentes cariados, perdidos e obturados) e critérios da Organização Mundial da Saúde (OMS) para diagnóstico de cárie e perda dentária^4,7-9^.

Estudo de Ferreira et al.^11^ investigou a evolução da cárie dentária e do edentulismo em idosos brasileiros entre 2003 e 2023 e apontou que o índice CPOD médio caiu de 27,6 para 23,5 e o edentulismo — perda total dos dentes — reduziu de cerca de 53% para 36%, concentrando-se em pessoas brancas e com maior escolaridade^11^. Contudo, há poucos estudos com análises agregadas que incorporem variáveis dos serviços de saúde e as diferenças de tendência na trajetória de vida, desde a vida adulta até os idosos^6^, comparando-se os dois grupos. Nesse sentido, o presente estudo buscou analisar as tendências da perda dental e severidade da cárie dentária entre adultos e idosos nos municípios participantes dos três Inquéritos Nacionais de Saúde Bucal (SB Brasil – 2003, 2010 e 2023), numa estratégia de análises intramunicípio e de controle de variáveis contextuais ao longo do tempo. Além disso, buscou identificar as diferenças entre as faixas etárias e variáveis associadas nos aspectos socioeconômicos e de serviços de saúde.

## MÉTODOS

Trata-se de um estudo ecológico com abordagem quantitativa, baseado em dados secundários provenientes dos SB Brasil 2003, 2010 e 2023^7-9^. Foram incluídos na análise os 43 municípios que participaram de todas as edições do inquérito, garantindo a comparabilidade temporal (Quadro 1). O tamanho da amostra foi determinado pela disponibilidade dos dados provenientes dos inquéritos nacionais de saúde bucal realizados no período analisado, resultando na inclusão dos referidos municípios com informações completas e comparáveis ao longo do tempo.

**Quadro 1:** Municípios selecionados no estudo que participaram dos levantamentos epidemiológicos nos anos de 2003, 2010 e 2023.

Capitais	Municípios de grande porte em 2023 (Unidade Federativa)	Municípios de médio porte em 2023 (Unidade Federativa)
Aracaju (SE) Belém (PA) Belo Horizonte (MG) Boa Vista (RR) Brasília (DF) Campo Grande (MS) Cuiabá (MT) Curitiba (PR) Florianópolis (SC) Fortaleza (CE) Goiânia (GO) João Pessoa (PB) Macapá (AP) Maceió (AL) Manaus (AM) Natal (RN) Palmas (TO) Porto Alegre (RS0 Porto Velho (RO) Recife (PE) Rio Branco (AC) Rio de Janeiro (RJ) Salvador (BA) São Luís (MA) São Paulo (SP) Terezina (PI) Vitória (ES)	Ananindeua (PA) Aparecida de Goiânia (GO) Blumenau (SC) Cametá (PA) Duque de Caxias (RJ) Itajaí (SC) Luziânia (GO) Parauapebas (PA) Santarém (PA) Várzea Grande (MT)	Anápolis (GO) Cáceres (MT) Corumbá (MS) Cruzeiro do Sul (AC) Parintins (AM) Tefé (AM)

Pequeno porte: 5.001 a 20 mil habitantes; médio porte: 20.001 a 100 mil habitantes; grande porte: acima de 100 mil habitantes.

Fonte: Dados do SB Brasil 2003, 2010 e 2023.

O foco do estudo está na severidade da cárie e da perda dentária. Os desfechos considerados foram as médias municipais do índice CPOD (número médio de dentes cariados, perdidos e obturados por indivíduo) e, especificamente, do seu componente "P", que representa os dentes perdidos. As análises foram estratificadas por faixa etária, contemplando adultos (35–44 anos) e idosos (65–74 anos). A participação % do componente "P" no CPOD também foi analisada. Os bancos de dados nacionais utilizados são produto da Pesquisa SB Brasil 2003, 2010 e 2023, cujos resultados são atualmente um banco público de acesso livre e gratuito. Cabe destacar que os dados do SB Brasil 2003, 2010 e 2023 decorrem de delineamentos amostrais complexos e independentes. No presente estudo, os desfechos foram operacionalizados como proporções agregadas por município. Como os pesos amostrais individuais não estão disponíveis para o SB Brasil 2003, optou-se por uma abordagem ecológica e comparativa, sem ponderação individual.

Para avaliar as tendências e o padrão de variabilidade dos desfechos, foram criados gráficos do tipo *spaghetti*, acompanhados de uma curva média representando o comportamento geral nos três anos avaliados. Para cada desfecho e em cada ano, apresentaram-se a média, o desvio-padrão, os valores mínimo e máximo e o intervalo de confiança de 95% da média. Foram consideradas como covariáveis a taxa de analfabetismo (%), a cobertura populacional das equipes de Saúde Bucal (eSB) no Sistema Único de Saúde (SUS), a cobertura por planos odontológicos privados (% da população com plano) e a situação do município (capital ou não capital).

Os dados relativos à cobertura das eSB (equipes 40 horas, já incluídas eSB Quilombolas/Assentados, modalidade I e II) para 2003, 2010 e 2023 foram coletados no site eGestor Atenção Básica, no endereço https://egestorab.saude.gov.br. A taxa de cobertura de plano de saúde exclusivamente odontológico foi obtida por meio do sistema TabNet da Agência Nacional de Saúde Suplementar (ANS), no endereço https://www.ans.gov.br/anstabnet/cgi-bin/dh?dados/tabnet_tx.def.

A taxa de analfabetismo da população com 15 anos ou mais foi obtida de acordo com os dados do Atlas do Desenvolvimento Humano no Brasil (Atlas DH), com base nos microdados dos Censos Demográficos do Instituto Brasileiro de Geografia e Estatística (IBGE). Foram utilizados os dados dos Censos Demográficos de 2000, 2010 e 2022, no endereço https://www.ipeadata.gov.br/Default.aspx, correspondendo aos anos mais próximos dos inquéritos analisados (2003, 2010 e 2023).

Para avaliar as associações entre essas variáveis explicativas e os desfechos em três momentos distintos (2003, 2010 e 2023), em cada estrato etário, foram ajustados modelos de Equações de Estimação Generalizadas (*Generalized Estimating Equations –* GEE) conforme proposto por Liang e Zeger^12^.

Os modelos foram especificados com função de ligação identidade e distribuição normal (gaussiana), apropriadas a desfechos contínuos. Diferentes estruturas de correlação de trabalho foram testadas (*independence*, *AR(1)*, *unstructured* e *exchangeable*), sendo selecionada a estrutura *exchangeable* por pressupor correlação constante entre tempos e apresentar melhor estabilidade numérica e convergência. As variâncias dos estimadores foram obtidas por meio do estimador robusto tipo *sandwich*, garantindo inferência consistente mesmo sob eventual especificação incorreta da correlação. Todas as análises foram conduzidas no software R (versão 4.4.3) (*R Core Team*, 2025), adotando-se nível de significância de 5%^13^. O relato do estudo seguiu as recomendações do checklist STROBE (*Strengthening the Reporting of Observational Studies in Epidemiology* — Fortalecendo o Relato de Estudos Observacionais em Epidemiologia).

### Declaração de disponibilidade de dados:

Todo o conjunto de dados que dá suporte aos resultados deste estudo está disponível mediante solicitação ao autor correspondente. O conjunto de dados não está publicamente disponível devido à integração de bancos de dados realizados pelos autores.

## RESULTADOS

No conjunto dos 43 municípios analisados, a redução do CPOD, bem como do componente Perdido, foi expressiva no período. Contudo, dois municípios apresentaram tendência divergente: Santarém, no Pará, e Cárceres, no Mato Grosso, onde este indicador entre os adultos aumentou e estabilizou, respectivamente (Quadro 1). Entre os idosos, apenas Aparecida de Goiânia, no Centro-Oeste, também apresentou tendência divergente de aumento em relação aos demais municípios. Na análise visual da curva de tendência, observa-se que a queda foi maior entre os adultos, comparativamente aos idosos (Figura 1).

**Figura 1:** Tendência das médias do índice CPOD e do componente "P" (dentes perdidos) nos anos de 2003, 2010 e 2023, segundo as faixas etárias de 35-44 anos (painéis superiores) e 65–74 anos (painéis inferiores), entre os municípios participantes das três edições do Inquérito Nacional de Saúde Bucal (n=43).

Fonte: Dados do SB Brasil 2003, 2010 e 2023.

A análise longitudinal evidenciou uma tendência decrescente nos indicadores de saúde bucal, entre 2003 e 2023, para os adultos (Tabela 1). Neste grupo de 35 a 44 anos, a média do índice CPOD reduziu de 19,17 (IC95% 18,37–19,98) em 2003 para 16,67 (IC95% 15,90–17,43) em 2010 e 10,32 (IC95% 9,27–11,37) em 2023, indicando reduções sucessivas e intervalos de confiança não sobrepostos entre os três períodos. O componente "P" (dentes perdidos) apresentou comportamento semelhante, com declínio de 12,02 (IC95% 10,99–13,05) em 2003 para 8,58 (IC95% 7,71–9,46) em 2010 e 3,69 (IC95% 2,83–4,54) em 2023, correspondendo a uma queda acumulada superior a 65% no período (Tabela 1).

**Tabela 1:** Média, desvio-padrão e intervalo de confiança de 95% para o CPOD, componente "P" (dentes perdidos), e % Perdidos no CPOD entre adultos (35–44 anos) e idosos (65–74 anos) em municípios acompanhados nas três edições do SB Brasil (2003, 2010 e 2023).

Ano		Média	d.p.	% P no CPOD	Mínimo	Máximo	IC95%
CPOD 35–44 anos							
	2003	19,17	2,70	-	9,94	23,69	18,37–19,98
	2010	16,67	2,56	-	10,17	22,33	15,90–17,43
	2023	10,32	3,51	-	2,50	22,20	9,27–11,37
Dentes perdidos 35–44 anos							
	2003	12,02	3,45	62.70	3,82	20,11	10,99–13,05
	2010	8,58	2,93	51.47	1,95	15,17	7,71–9,46
	2023	3,69	2,87	35.76	0,50	17,80	2,83–4,54
CPOD 65–74 anos							
	2003	27,17	3,20	-	16,14	32,0	26,21–28,12
	2010	26,66	1,92	-	21,63	30,8	26,08–27,23
	2023	23,91	3,11	-	15,00	32,0	22,98–24,84
Dentes perdidos 65–74 anos							
	2003	24,98	4,22	91.94	10,71	32,00	23,71–26,24
	2010	24,86	3,00	93.25	18,78	30,44	23,97–25,76
	2023	20,77	4,22	86.87	11,02	32,00	19,51–22,03

Fonte: SB Brasil 2003, 2010 e 2023.

Entre os idosos de 65 a 74 anos, observou-se estabilidade entre 2003 e 2010, tanto para o CPOD quanto para o componente "P", cujos intervalos de confiança se sobrepõem amplamente (CPOD 27,17 [IC95% 26,21–28,12] em 2003 e 26,66 [IC95% 26,08–27,23] em 2010; "P": 24,98 [IC95% 23,71–26,24] e 24,86 [IC95% 23,97–25,76], respectivamente). Apenas em 2023 nota-se redução significativa, com médias de 23,91 (IC95% 22,98–24,84) para o CPOD e 20,77 (IC95% 19,51–22,03) para o componente "P" (Tabela 1). Esses resultados indicam avanços consistentes na manutenção dentária ao longo das duas décadas, embora o ritmo de melhora tenha sido maior entre os adultos. Quanto à participação proporcional dos dentes perdidos no CPOD, observa-se que esta reduz fortemente no grupo de adultos entre 2003 e 2023, mas não na mesma magnitude entre os idosos. O componente "P" representava 62,7% do CPOD em 2003, passando para 35,76% em 2023. Já entre os idosos, este representava 91,94% do CPOD em 2003 e reduziu discretamente para 86,87% em 2023 (Tabela 1).

A modelagem por GEE permitiu avaliar a influência de fatores contextuais sobre os desfechos de saúde bucal, considerando a correlação intramunicípio ao longo do tempo. Entre os adultos (35–44 anos), o modelo para o CPOD indicou associação positiva entre a taxa de analfabetismo e a média do índice (β=0,309; p<0,001), revelando que municípios com maior proporção de analfabetos apresentaram piores indicadores de CPOD. Em contrapartida, a cobertura das eSB mostrou associação negativa e significativa (β=-0,040; p=0,011), sugerindo efeito protetor da expansão da saúde bucal na atenção básica sobre a severidade da cárie e perda dentária entre os adultos. A cobertura por planos odontológicos privados também apresentou associação negativa significativa (β=-4,527; p<0,001), indicando que maior acesso a serviços privados de odontologia se relaciona a menores médias do índice CPOD (Tabela 2).

**Tabela 2:** Resultados de modelos GEE para fatores associados à tendência de redução do CPOD e de dentes perdidos (média) entre adultos (35–44 anos) e idosos (65–74 anos) em municípios (n=43) participantes de todos os Inquéritos Nacionais SB Brasil (2003, 2010 e 2023).

Variável		Associação estimada (β)	Erro-padrão	Estatística do teste	p-valor
CPOD 35–44 anos					
	(Intercept)	15,350	1,341	130,994	<0,001
	Analf	**0,309**	**0,084**	**13,371**	**<0,001**
	Cob_esb	**-0,040**	**0,011**	**12,146**	**<0,001**
	Cob_ps	**-4,527**	**0,640**	**49,980**	**<0,001**
	Capital	1,541	0,980	2,476	0,116
Dentes perdidos 35–44 anos					
	(Intercept)	6,976	1,209	33,282	<0,001
	Analf	**0,507**	**0,079**	**41,555**	**<0,001**
	Cob_esb	**-0,046**	**0,011**	**16,747**	**<0,001**
	Cob_ps	**-3,574**	**0,469**	**58,104**	**<0,001**
	Capital	0,968	0,853	1,287	0,257
CPOD 65–74 anos					
	(Intercept)	26,863	0,822	928,238	<0,001
	Analf	0,043	0,066	0,432	0,511
	Cob_esb	-0,017	0,010	2,611	0,106
	Cob_ps	**-2,586**	**0,447**	**33,476**	**<0,001**
	Capital	0,404	0,706	0,327	0,567
Dentes perdidos 65–74 anos					
	(Intercept)	24,665	1,009	586,185	<0,001
	Analf	0,063	0,081	0,605	0,437
	Cob_esb	-0,013	0,013	0,924	0,336
	Cob_ps	**-3,766**	**0,758**	**24,705**	**<0,001**
	Capital	0,356	0,845	0,178	0,673

Em negrito para a significância estatística.

Fonte: SB Brasil 2003, 2010 e 2023.

Para o componente "P" (dentes perdidos) no mesmo grupo etário, observou-se padrão semelhante. A taxa de analfabetismo apresentou associação positiva e significativa (β=0,507; p<0,001), reforçando o papel do nível educacional nas desigualdades em saúde bucal. A cobertura das eSB manteve associação negativa (β=-0,046; p<0,001), indicando que maior cobertura está relacionada à redução no número médio de dentes perdidos. A cobertura por planos odontológicos privados também mostrou associação negativa significativa (β=-3,574; p<0,001), sugerindo que o acesso a serviços odontológicos privados contribui adicionalmente para menores perdas dentárias médias entre os indivíduos de 35–44 anos. De forma geral, o estudo aponta que melhores níveis educacionais e menor cobertura pública e privada estão associados a desfechos mais desfavoráveis, enquanto a ampliação das equipes de Saúde Bucal exerce efeito protetor neste grupo (Tabela 2).

Entre os idosos (65–74 anos), os coeficientes estimados apresentaram menor magnitude e significância, indicando atenuação dos efeitos contextuais e maior estabilidade dos indicadores ao longo do tempo. Para o CPOD, o analfabetismo não se associou significativamente ao desfecho (β=0,043; p=0,511), e a cobertura das eSB apresentou associação negativa não significativa (β=-0,017; p=0,106). Já a cobertura por planos odontológicos privados apresentou associação significativa (β=-2,586; p<0,001). Resultados semelhantes foram observados para o componente "P", no qual a taxa de analfabetismo não se associou significativamente ao número médio de dentes perdidos (β=0,063; p=0,437), enquanto a cobertura das eSB manteve associação negativa não significativa (β=-0,013; p=0,336). Por outro lado, a cobertura por planos odontológicos privados manteve associação negativa e estatisticamente significativa com o número médio de dentes perdidos (β=-3,766; p<0,001). A condição de capital não influenciou nenhum dos modelos ajustados (Tabela 2).

## DISCUSSÃO

O presente estudo confirma a tendência de declínio expressivo da perda dentária e do CPOD entre 2003 e 2023 — maior entre 2010 e 2023, especialmente na população adulta — e destaca neste grupo o papel protetor da escolaridade, ampliação da cobertura das eSB e do acesso a planos odontológicos privados. As desigualdades socioeconômicas, expressas pela taxa de analfabetismo, mantêm influência importante sobre os resultados de saúde bucal, sobretudo nas faixas etárias mais jovens.

Entre os idosos, não houve efeito estatisticamente significativo da taxa de analfabetismo, indicando que os impactos cumulativos do ciclo de vida precisam ser melhor investigados. Sugere-se que características desse ciclo da vida e da trajetória assistencial tendem a se sobrepor às condições educacionais atuais. Outras prováveis explicações estão relacionadas às características do envelhecimento no Brasil, que apresenta graves desigualdades, de natureza psíquica, física e mental^14^.

Estudos têm sido conduzidos para explorar as características específicas da perda dentária entre idosos no Brasil e em outros países^15-17^. Sabe-se que os idosos são especialmente vulneráveis à rápida deterioração da saúde oral, em inglês denominada ROHD (*Rapid Oral Health Deterioration*), devido a fatores como comorbidades crônicas, polifarmácia, restaurações dentárias extensas e desafios socioeconômicos relacionados à idade^15^.

A perda dentária, particularmente o edentulismo total, é um indicador robusto da condição geral de saúde oral, persistindo como um problema de saúde pública global significativo, especialmente entre a população idosa^18^. A perda dentária não se restringe à cavidade oral, tendo repercussões significativas na saúde sistêmica, especialmente em idosos, na qual está associada ao aumento da incidência de síndromes geriátricas. Indivíduos edêntulos ou com perda dentária parcial são mais propensos a estar desnutridos ou em risco de desnutrição^19^.

Cabe destacar que o envelhecimento populacional é um fenômeno global e multifacetado, resultado direto do aumento da expectativa de vida e da redução das taxas de fecundidade^20^. Embora represente uma conquista histórica da humanidade, esse processo traz consigo desafios complexos para os sistemas de saúde, previdência e assistência social. Na saúde bucal, as implicações do envelhecimento são ainda mais visíveis, pois as doenças bucais, cumulativas ao longo da vida, resultam frequentemente em perda dentária, que, por sua vez, compromete funções essenciais como mastigação, deglutição e fala, além de impactar a estética e a autoestima dos indivíduos^20-23^. Embora a prevalência padronizada por idade tenha diminuído em muitos países desenvolvidos nas últimas décadas, o crescimento absoluto da população idosa gerou um aumento no número total de pessoas com perda dentária severa, como demonstrado pelos dados do Global Burden of Disease entre 1990 e 2019^20^.

Também como o estudo de Ferreira et al.^11^, o presente estudo encontrou redução significativa no número de dentes perdidos entre 2023 e 2003, mas não observada entre 2010 e 2003. Em outras palavras, os dados confirmaram a análise do agregado dos 43 municípios do presente estudo. Contudo, cabe destacar que a redução foi desigual, beneficiando principalmente grupos mais escolarizados e brancos^11^, ou seja, fatores como baixa escolaridade, pobreza, comorbidades crônicas, tabagismo e dieta inadequada têm sido identificados como determinantes de risco de edentulismo em estudos multicêntricos envolvendo países como China, Índia, México e África do Sul^23^.

Esse cenário reforça a urgência de políticas públicas que integrem a saúde bucal aos modelos de atenção voltados ao envelhecimento, pois a perda dentária não pode ser vista como um resultado inevitável do envelhecimento. A prevalência média global de perda dentária é de 7% (350 milhões de pessoas)^24^, o que reforça a necessidade de intervenções preventivas desde as primeiras décadas de vida, bem como ações de promoção da saúde para redução da perda dental entre idosos^25^. Os estudos reconhecem a importância da APS na saúde bucal^25,26^, pois o aumento da cobertura de eSB no modelo da ESF tem sido relacionado à redução de indicadores de perda dentária, convergindo com o achado do presente estudo de redução do componente "P" (perdido) apenas entre os adultos. Ainda assim, estudo apontou que em cidades com menor Índice de Desenvolvimento Humano (IDH) e menor cobertura de eSB houve maior proporção de extrações dentárias, com menor valor de IDH^27^. Iniciativa recente de incorporar indicador que incentiva a redução da porcentagem de exodontias em relação aos demais procedimentos na atenção primária busca intervir de forma resolutiva no início da história natural da doença (cárie e doença periodontal), privilegiando procedimentos menos mutiladores (preventivos e curativos) em detrimento de extrações^28^.

Fatores associados a melhor saúde bucal em idosos incluem acesso regular a serviços odontológicos, e o efeito protetor do seguro é mais forte entre aqueles com status socioeconômico mais elevado e estilos de vida mais saudáveis. O presente estudo apontou efeito positivo entre idosos com posse de plano odontológico privado e menor perda dental. Contudo, a posse de um plano odontológico privado por si só não elimina as disparidades; a educação, o rendimento e os comportamentos de saúde continuam a ser determinantes importantes^29^.

Além disso, estudo recente apontou que a ausência de plano odontológico foi significativamente associada a uma maior prevalência de edentulismo, mesmo após ajustes para idade, escolaridade e estilo de vida^30^. A ausência de plano odontológico privado provavelmente reflete tanto o menor poder aquisitivo quanto o menor acesso a cuidados odontológicos preventivos e restauradores, aumentando o risco de perda dentária^31^.

Um estudo transversal da mesma base populacional utilizada neste estudo evidenciou uma alta prevalência de edentulismo total entre idosos brasileiros (31,7%), revelando um importante problema de saúde pública com repercussões estéticas, funcionais e psicossociais. Foi realizada uma análise multivariada, que demonstrou que a perda dentária total está associada a condições socioeconômicas desfavoráveis, baixa escolaridade e ausência de plano odontológico, refletindo desigualdades no acesso à prevenção e ao tratamento. Além disso, fatores comportamentais como tabagismo, consumo de bebidas açucaradas e sedentarismo mostraram-se significativamente relacionados à condição, reforçando o caráter multifatorial do edentulismo^32^. Esses achados sustentam a necessidade de políticas públicas integradas que promovam hábitos saudáveis e ampliem o acesso aos cuidados odontológicos, especialmente entre os grupos mais vulneráveis da população idosa^17^.

O relatório internacional publicado em 2022 pela Organização Internacional da Saúde destaca a relevância global da saúde bucal no envelhecimento, alinhando-se à iniciativa da "Década do Envelhecimento Saudável (2021–2030)" da Organização das Nações Unidas (ONU), que reconhece a saúde oral como indicador essencial da saúde geral em idosos. As doenças bucais, de caráter crônico e cumulativo, apresentam alta prevalência nas faixas etárias avançadas, especialmente o edentulismo, cuja média global é de 22,7% entre pessoas acima de 60 anos, com variações regionais significativas — por exemplo, a Europa apresenta as taxas mais elevadas (31,3%). A doença periodontal grave também atinge seu pico por volta dos 60 anos, e o envelhecimento populacional tende a aumentar sua carga mundial. O documento ressalta ainda que as desigualdades sociais e econômicas agravam a perda dentária, afetando desproporcionalmente idosos frágeis e institucionalizados. Como exemplo de política eficaz, o Japão é citado com a "Campanha 8020", voltada à preservação de 20 dentes naturais aos 80 anos de idade, reforçando que a prevenção contínua e o cuidado bucal ao longo da vida resultam em melhor nutrição, autonomia e integração social na velhice^24,33^.

Portanto, estudos que incorporem indicadores sistêmicos de saúde, socioeconômicos e de saúde bucal contribuem para identificar, diagnosticar e tratar condições que podem levar à perda dentária^16^. É preciso avançar em análises de fatores que produzem a ROHD^15,16^, associados à perda dentária entre idosos brasileiros. Estudos futuros desta natureza poderão fornecer *insights* baseados em evidências que possam ajudar os formuladores e implementadores de políticas, bem como os profissionais de saúde bucal brasileiros, a detectar e apoiar melhor aqueles em risco de rápida deterioração da saúde oral.

Neste sentido, recomendações elaboradas pelo *European College of Gerodontology* enfatizam que muitos profissionais de saúde não estão preparados para identificar ou manejar problemas bucais em idosos e, ainda, que barreiras estruturais — incluindo baixa cobertura pública, falta de integração entre odontologia e atenção médica e dificuldades de acesso — contribuem para o subdiagnóstico e a falta de tratamento^33^.

O presente estudo, ao utilizar dados agregados, não permitiu uma análise mais aprofundada da qualidade de vida dos idosos, o que se configura como uma limitação. Além disso, os dados do SB Brasil 2003, 2010 e 2023 decorrem de delineamentos amostrais complexos e independentes.

No presente estudo, os desfechos foram operacionalizados como proporções agregadas por município, e o objetivo analítico foi estimar variações temporais relativas e diferenças entre grupos de municípios. Reconhece-se que seria possível calcular proporções ponderadas de acordo com os pesos individuais. Contudo, os pesos amostrais não estão disponíveis para o SB Brasil 2003. Diante disso, optou-se por uma abordagem ecológica e comparativa, reconhecendo essa decisão como uma limitação metodológica do estudo. Outra limitação foi que a amostra final compreendeu apenas municípios presentes nos 3 inquéritos (n=43), o que resultou em maior representação de capitais e municípios de grande porte. Para mitigar possíveis desequilíbrios, incluímos uma variável indicador "ser capital" nos modelos ajustados. Novos estudos que aprofundem as características dos idosos brasileiros que têm levado à maior perda dental são indicados, sendo este grupo mais refratário ao processo histórico-social que tem reduzido a prevalência desse agravo nos demais grupos.

Este estudo confirmou a tendência de declínio no índice CPOD e de perda dentária entre adultos e idosos. Características como menor analfabetismo, cobertura de eSB e possuir plano privado odontológico se mostraram como aspectos favoráveis a favor desta queda entre os adultos. Já entre os idosos, as associações foram menos evidentes e significativa apenas para posse de plano odontológico privado, indicando atenuação dos efeitos contextuais e estabilidade dos indicadores ao longo do tempo.
